# The Three Faces of Riboviral Spontaneous Mutation: Spectrum, Mode of Genome Replication, and Mutation Rate

**DOI:** 10.1371/journal.pgen.1002832

**Published:** 2012-07-26

**Authors:** Libertad García-Villada, John W. Drake

**Affiliations:** Laboratory of Molecular Genetics, National Institute of Environmental Health Sciences, Research Triangle Park, North Carolina, United States of America; Uppsala University, Sweden

## Abstract

Riboviruses (RNA viruses without DNA replication intermediates) are the most abundant pathogens infecting animals and plants. Only a few riboviral infections can be controlled with antiviral drugs, mainly because of the rapid appearance of resistance mutations. Little reliable information is available concerning i) kinds and relative frequencies of mutations (the mutational spectrum), ii) mode of genome replication and mutation accumulation, and iii) rates of spontaneous mutation. To illuminate these issues, we developed a model *in vivo* system based on phage Qß infecting its natural host, *Escherichia coli*. The Qß *RT* gene encoding the Read-Through protein was used as a mutation reporter. To reduce uncertainties in mutation frequencies due to selection, the experimental Qß populations were established after a single cycle of infection and selection against *RT* ^−^ mutants during phage growth was ameliorated by plasmid-based *RT* complementation *in trans*. The dynamics of Qß genome replication were confirmed to reflect the linear process of iterative copying (the stamping-machine mode). A total of 32 *RT* mutants were detected among 7,517 Qß isolates. Sequencing analysis of 45 *RT* mutations revealed a spectrum dominated by 39 transitions, plus 4 transversions and 2 indels. A clear template•primer mismatch bias was observed: A•C>C•A>U•G>G•U> transversion mismatches. The average mutation rate per base replication was ≈9.1×10^−6^ for base substitutions and ≈2.3×10^−7^ for indels. The estimated mutation rate per genome replication, μ*_g_*, was ≈0.04 (or, per phage generation, ≈0.08), although secondary *RT* mutations arose during the growth of some *RT* mutants at a rate about 7-fold higher, signaling the possible impact of transitory bouts of hypermutation. These results are contrasted with those previously reported for other riboviruses to depict the current state of the art in riboviral mutagenesis.

## Author Summary

Viral disease is a subject of major concern in public health. Diseases produced by riboviruses (RNA viruses *sensu stricto*) represent a special urgency, because these viruses display an exceptional capability to generate resistance mutations against antiviral drugs. Unfortunately, little is known about the rate and nature of spontaneous mutation in riboviruses. Thus, characterization of their mutation process may be helpful in the development of improved ways to counteract riboviral diseases. In this study, we investigated the mutation process *in vivo* of a model ribovirus, the bacteriophage Qß, focusing on three key aspects: i) the kinds and relative frequencies of mutations, ii) the mode of genome replication, and iii) the rate of spontaneous mutation. Our results, combined with other information about riboviral mutagenesis, depict a ribovirus mutation spectrum largely dominated by transitions, a predominantly linear mode of genome replication, and a mutation rate per genome replication on the order of 0.04 for bacteriophages and plant viruses but perhaps an order of magnitude higher for mammalian riboviruses.

## Introduction

Riboviruses (RNA viruses with no DNA replication intermediates) infect organisms from prokaryotes to higher eukaryotes and frequently cause deadly diseases. The mortality, morbidity, and economic burden of ribovirus-borne diseases strongly impact human society, especially in developing countries where neither sanitation nor treatment may be adequate [1]. Although extensive efforts have focused on developing countermeasures to prevent or treat riboviral diseases, only a few of these diseases can be effectively controlled by vaccination or antiviral drugs. In addition, control or eradication of riboviral diseases is soon balanced by the emergence of new riboviral pathogens or treatment-resistant strains of old ones (reviewed in [2]). Thus, we seek to understand which special features of these viruses contribute to their success. One key feature is their high mutation rate (reviewed in [3]).

Although the evolutionary forces driving high riboviral mutation rates remain unclear (reviewed in [1]), three mechanistic factors play important roles: the higher error-insertion rates of RNA replicases compared to DNA replicases, the lack of proofreading activity in RNA replicases, and the nonexistence of post-replicative RNA mismatch repair. The estimated mean rate per infection cycle is about 1.3 for several common single-stranded RNA (ssRNA) human pathogens [4], roughly 0.1 for ssRNA tobacco viruses [5], and 0.03 for the double-stranded RNA (dsRNA) bacteriophage φ6 [6]. Unfortunately, most of these estimates were based on studies in which small, potentially unrepresentative sequences were used as mutation reporters. In some cases, estimated rates in excess of 1 per infection cycle are probably incompatible with viability [7]. A further problem is the scarce information on the mode (linear, exponential, or mixed) by which riboviruses replicate their genomes within the host cell. Distinct modes of genome replication impact the pattern of intra-cell mutation accumulation in the riboviral genome (and hence the mutation rate per infection cycle) differently. The only two empirical studies published to date on riboviral replication strategy, one conducted with the phage φ6 [8] and the other with the ssRNA turnip mosaic virus [9], suggest that riboviruses replicate their genome mostly in a linear fashion, but further results are needed based on other riboviral systems.

In addition, there are limited data on the kinds and relative frequencies of spontaneous mutations (the mutation spectrum) in riboviruses, again a reflection of mutation reporters that do not sufficiently sample the genome. Only three spontaneous mutation spectra based on a cognate riboviral gene of adequate size are available and, unfortunately, none seems to be fully illustrative. The tobacco mosaic virus rate and spectrum [10] were derived under conditions of multiple sequential infections. The tobacco etch potyvirus spectrum [11] probably contains a large fraction of mutations resulting from methodological manipulations rather than from virus replication errors. Finally, the phage φ6 spectrum [6] was obtained from a mutation-accumulation experiment in the absence of gene complementation *in trans*, which tends to discriminate against strongly deleterious mutations.

A complete portrait of spontaneous mutagenesis in riboviruses is important not only for understanding their prevalence but also for improving ways to prevent and to treat riboviral diseases. For instance, accurate information on riboviral mutation kinds and rates may facilitate the creation of more stable attenuated vaccines [12]. Similarly, it seems likely that antiviral treatments based on mutagenic base analogs may prove to be more effective if the base analogs specifically increase the rate of those errors that riboviral replicases already generate most frequently. Although pathway-directed mutagenesis is unlikely to prevent the appearance of riboviral resistance to specific base analogs, it may enlighten the development of more efficient combinatory therapies [13] and at least slow disease progression, thus enhancing the immune response.

The main aims of the present study were to characterize the mutation spectrum, to determine the mode of genome replication, and to estimate the spontaneous mutation rate of a ribovirus using the bacteriophage Qß as an experimental model. Qß has been well characterized physiologically [*e.g.*], [ 14]–[17], physiochemically [*e.g.*], [ 18]–[21], structurally [*e.g.*], [ 22]–[27], and molecularly [*e.g.*], [ 28]–[32]. It is a linear (+)-strand ssRNA phage whose natural host is *Escherichia coli*, although it can also propagate in other gram-negative bacteria with an F pilus. Its 4217-nt long genome is organized in three cistrons that encode (from 5′ to 3′) the A2 or Maturation protein, which mediates both the binding of Qß to the host and post-replicative host lysis; the Coat protein and its elongated A1 or Read-Through (RT) protein, which is required for Qß capsid assembly and for host infection; and the catalytic ß subunit of the Qß replicase. (*RT* is translated when a ribosome incorporates tryptophan at the natural UGA stop codon of the Coat-coding gene at a frequency of ≈3% [33].) Qß's life cycle may be summarized as follows: i) the phage attaches to the F pilus of *E. coli* and the genome enters the cytoplasm; ii) cellular components translate the ß subunit of the phage replicase, which then polymerizes with four host subunits (the ribosomal protein S1, the translation elongation factors EF-Tu and EF-Ts, and the host factor HF) and binds the Qß genome; iii) the ß subunit copies the (+)-strand genome to produce a (−)-strand RNA that in turn is used as template to produce more (+) strands; iv) (+) strands serve as templates for the production of the phage proteins; v) 40–60 minutes after infection, by which time the host cell is filled with phage particles, partially assembled virions, and phage-specific side products, the cell lyses, releasing (10–40)×10^3^ particles of which only 10–50% are infectious (reviewed in [14], [34]).

Here, we used the gene encoding the RT protein (excluding the portion that encodes the Coat protein) as an *in vivo* mutation reporter. Selection against *RT* ^−^ mutants was ameliorated by using a complementing system *in trans* based on a plasmid that encodes the entire Coat/RT mRNA with the natural UGA stop codon replaced with a TGG tryptophan codon [35]. To further reduce the effect of selection, the experimental Qß populations were established after a single cycle of infection. We assessed the Qß genome mutation rate (μ*_g_*) in three different ways: i) a forward-mutation test in which mutants carrying phenotypically detectable *RT* mutations were isolated and sequenced and μ*_g_* was estimated from the frequency of observed nonsense mutations and indels; ii) single-burst reversion tests in which two different *RT* ^−^ mutants were employed (one carrying a single-base substitution and the other a four-base insertion) and μ*_g_* was estimated from the corresponding reversion rates; and iii) a phenotype-blind forward-mutation test in which some first-generation progeny of the *RT* mutants detected by the first method were isolated and sequenced and μ*_g_* was estimated from the frequency of all secondary *RT* mutations generated *de novo*. The distributions of *RT* ^+^ revertants observed in the reversion tests were used to infer the mode in which Qß replicates its genome, and the spontaneous Qß mutation spectrum was obtained from the *RT* mutations collected through the forward-mutation tests.

## Results

### Description of the System

**Figure 1:**
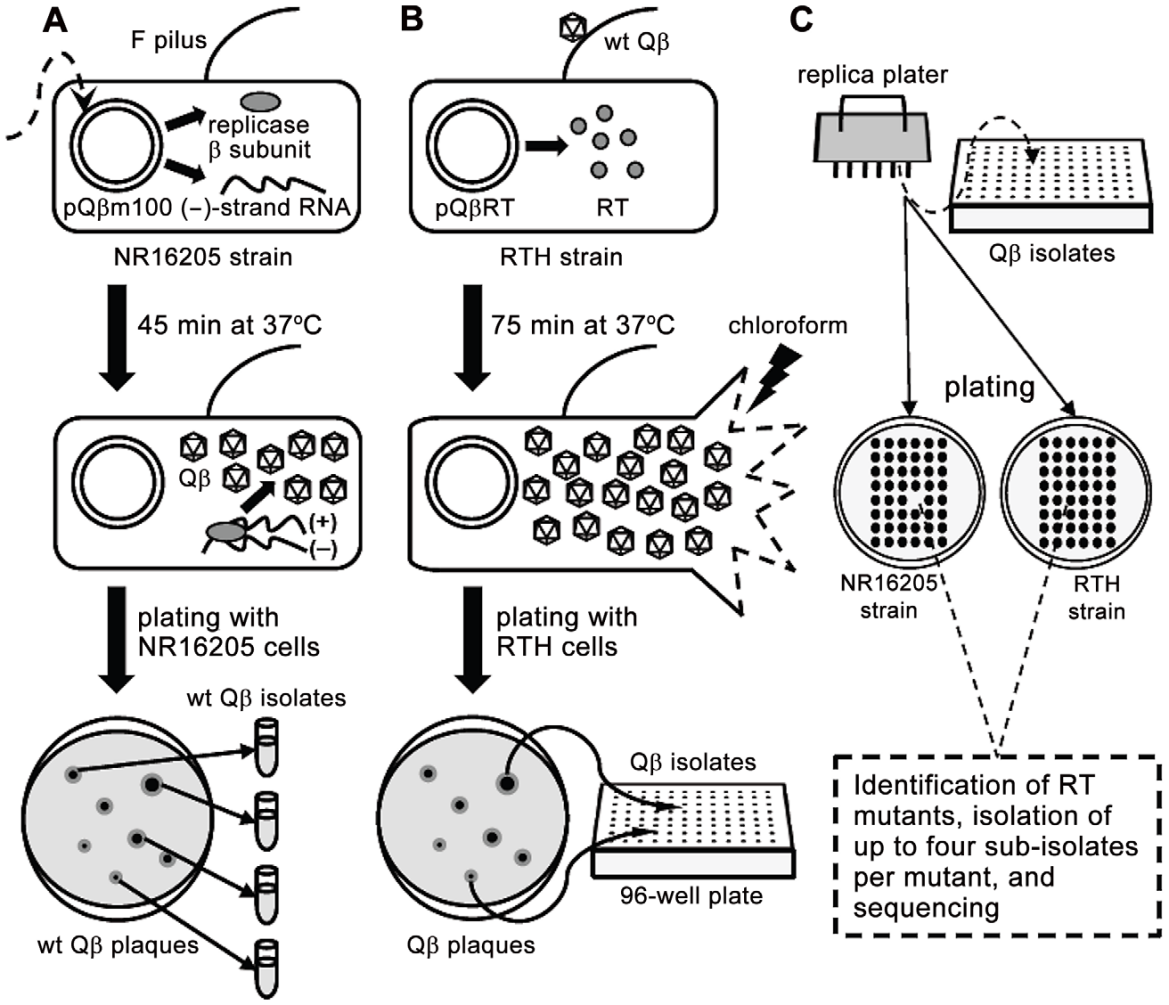
Schematic representation of the experimental system used to isolate *RT* mutants. A. *E. coli* NR16205 cells were transformed with pQßm100, which constitutively expresses low levels of Qß (−) strands. Some ß subunits of the Qß replicase are also transcribed from pQßm100 and, once translated, they replicate the (−) strands yielding (+) strands, which are infectious (*i.e.*, they produce Qß virions). Transformants were plated with NR16205 cells and incubated overnight to generate wt Qß plaques, which were individually isolated. B. *E. coli* RTH cells, which carry pQßRT that expresses RT constitutively, were infected with wt Qß. After one cycle of infection (75 min), chloroform was added to the experimental cultures to prevent further infections. Samples of the resulting lysates were plated with RTH cells, rendering Qß plaques that were independently harvested in 96-well plates. The use of RTH cells during these steps assures that any *RT* ^−^ mutant generated during the replication of wt Qß would be as able to produce a plaque as its wild-type siblings. C. Using a replica plater, the isolated Qß plaques were tested for the RT^−^ phenotype (impaired growth on non-complementing NR16205 cells but normal growth on RTH cells). Putative *RT* mutants were identified and sequenced, after which up to four sub-isolates per verified *RT* mutant were isolated and sequenced.

The basics of the experimental system and the strains used in this study are described in Figure 1 and Table 1, respectively.

**Table 1:** Strains.

System	Designation	Relevant properties	Source
Plasmids	pQßm100	Expresses wt Qß	DM
	pQßm100(RT2157/fs)	Expresses RT_IN_	DM
	pQßRT	Expresses RT and TMP^r^	DM
*E. coli*	NR10836	*ara thi* Δ*prolac* F′*prolac*	RS
	NR16205	NR10836 Tet^r^	RS
	RTH	NR10836+pQßRT	LGV
Qß	wt		pQßm100
	RT_IN_	+UUAA at 2157	pQßm100
	RT_SUB_	G1990A	LGV

Sources: DM = Donald Mills (SUNY Downstate Medical Center, Brooklyn, NY); RS = Roel Schaaper (National Institute of Environmental Health Sciences) [65]; LGV = this report.

### The Mutation Spectrum

Mutations arising in a mutation-reporter (target) sequence can be of two types. “Detectable” mutations are those that display the mutant phenotype when present as a single mutation. “Undetectable” mutations lack the mutant phenotype when present as a single mutation but may nevertheless be observed when they arise in the presence of a detectable mutation, in which case they are sometimes called “hitchhiker” mutations (and their detectable partner may be called a “driver” mutation). Sometimes, especially with mutants with equivocal phenotypes, no mutation is found in the target, reflecting either some imperfection in the screening method or a mutation elsewhere in the genome whose effect mimics that of the reference mutation; such isolates are thereafter included in the non-mutant total. Another distinction is often relevant: some mutations produce a fully mutant phenotype but others produce an intermediate phenotype (and are therefore often called “leaky” mutations or are said to produce a “weak” mutant phenotype). In this study, yet another dimension is added. Each Qß mutant originally isolated as requiring a helper host to generate a plaque or each of a number of non-mutant control plaques was re-plated and up to four next-generation plaques were harvested and sequenced. When all members of such a family contain the same mutation, we call it the “primary” mutation, and if some of the next-generation plaques contain additional mutations, we call them “secondary” mutations, which may arise when mutation rates are sufficiently high.

**Figure 2:**
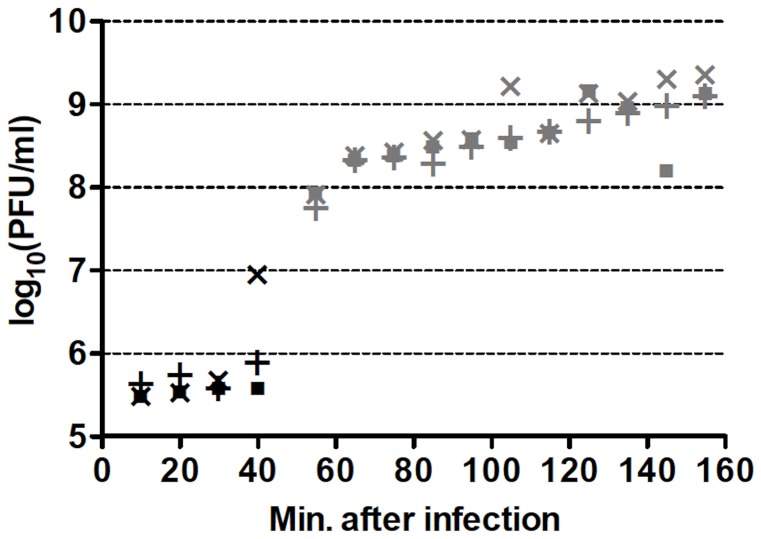
Representative one-step growth curves of wt Qß in RTH cells. Individual cultures are represented by different symbols while black and gray colors represent two different culture dilutions. PFU = plaque-forming units. Similar curves were obtained for each of the four wt Qß isolates used to generate one-step growth lysates.

One-step growth curves of wild-type (wt) Qß in RT-helper (RTH) cells, which complement *RT* ^−^ mutations, indicated that Qß requires ≈75 min to lyse an infected RTH cell (Figure 2). Thus, to limit the number of infection cycles to one before seeking *RT* mutants, RTH lysates were generated by adding chloroform 75 min after infection with wt Qß. Samples of these lysates were plated with RTH cells and the resulting plaques were harvested and tested for the RT^−^ phenotype (impaired growth on non-complementing NR16205 cells but normal growth on RTH cells). Among 7517 plaques tested in four independent experiments, 47 candidate *RT* mutants were recovered and sequenced. Of these, 30 contained at least one primary *RT* mutation (Table 2). (The 17 candidates with no primary *RT* mutation may have carried an RT^−^-mimicking mutation elsewhere in the genome or, because Qß grows better on RTH cells than on NR16205 cells, might have carried weak non-*RT* mutations and showed enhanced growth on RTH cells.) Most of the primary mutations were missense but two (one in mutant RT23 and one in RT37) were indels consisting of single-base additions. Two mutants each carried two primary mutations; in RT18, both were missense; in RT41, one was missense and the other was a synonym. Three mutants (RT10, RT40 and RT46) each carried a nonsense mutation that generated a stop codon; RT40 is a special case because it converted the leaky UGA codon that terminates the Qß Coat protein to a far less leaky UAA stop codon. In two cases, the primary mutation displayed at most a very weak phenotype upon re-plating: the primary mutation of RT27 was a synonym and that of RT33 was missense. These mutants are included in Table 2 and dependent calculations because their mutations could in principle produce a deleterious effect but would have no significant impact if disregarded. The 13 *RT* secondary mutations (Table 2) presumably arose sufficiently early during the growth of the screened plaques on RTH lawns to be detected among the next-generation progeny. They include 6 missense mutations and 7 synonyms, a ratio that deviates from the approximately 3.3∶1 ratio expected from the set of *RT* codons. Applying the binomial distribution, finding 6 missense among a total of 13 mutations has *P* = 0.014 and finding ≤6 has *P* = 0.018. This result presumably signals selection against *RT* mutations with strong effect during plaque growth on RTH lawns, consistent with the smaller burst sizes of RT_IN_ (a mutant Qß strain carrying a four-bases insertion in *RT*; see Table 1) than wt Qß in helper cells. (The average burst sizes of RT_IN_ and wt Qß are 328 and 847, respectively, estimated from three different one-step curves per phage type.)

**Table 2:** Spontaneous *RT* mutants and mutations.

Mutant	Mutation(s)	Position	Impact
RT1	G→U	383	Gly→Val
	(A→G)	(13)	(Ile→Val)
RT3	A→G	449	Asp→Gly
RT4	U→C	122	Leu→Pro
	(G→A)	(473)	(Gly→Asp)
RT5	G→A	383	Gly→Asp
	(U→G)	(283)	(Cys→Gly)
RT6	U→C	260	Leu→Pro
RT7	G→U	526	Asp→Tyr
RT10	G→A	249	Trp→Stop
RT14	U→C	388	Phe→Leu
	(U→C)	(411)	(Ile→Ile)
RT16	G→A	430	Ala→Thr
RT18	C→U	119	Ser→Phe
	A→G	527	Asp→Gly
RT20	U→C	460	Tyr→His
RT21	A→G	170	Tyr→Cys
RT23	+T	221–224	Frameshift
	(A→U)	(85)	(Thr→Ser)
RT25	A→G	449	Asp→Gly
RT26	U→C	11	Leu→Pro
RT27	U→C	261	Leu→Leu
	(A→G)	(478)	(Ile→Val)
RT29	U→C	389	Phe→Ser
RT31	G→A	367	Glu→Lys
	(U→C)	(21)	(Gly→Gly)
RT32	U→C	479	Ile→Thr
	(C→U)	(105)	(Pro→Pro)
RT33	G→A	382	Gly→Ser
	(C→U)	(442)	(Leu→Phe)
RT36	U→C	439	Ser→Pro
RT37	+T	106–107	Frameshift
RT40	G→A	2	Stop→Stop
	(C→U)	(213)	(Leu→Leu)
	(A→G)	(216)	(Lys→Lys)
RT41	U→C	24	Gly→Gly
	G→A	565	Ala→Thr
RT42	U→C	68	Ile→Thr
RT43	G→A	430	Ala→Thr
RT44	U→C	283	Cys→Arg
RT45	U→C	308	Leu→Pro
RT46	G→A	248	Trp→Stop
RT47	A→G	449	Asp→Gly

The 30 mutants were recovered from 7517 screened plaques and include two isolates (RT27 and RT33) in which the primary mutation had no RT^−^ phenotype, although their secondary mutation (or the combination of both) did. None of the other 17 candidates in the series RT1-RT47 displayed primary mutation, but two isolates, RT24 and RT35, had one secondary mutation each (C→U at position 18 and U→C at position 294, respectively). Altogether, 148 mutants were sequenced: [28 *RT* mutants carrying primary detectable mutations]+[4 next-generation isolates per each of these]+[2 next-generation isolates per each of four *RT* mutants (RT24, RT27, RT33 and RT35) that lacked detectable primary mutations]. The mutants were obtained in four different screens yielding 8, 5, 6 and 11 mutants in the order shown. Mutations in parentheses are secondary mutations. Mutant positions refer to the target sequence (1 through 591 including the UAG stop codon) that corresponds to Qß genomic positions 1743 through 2333.

Table 3 lists the kinds of mutations in the entire set of 45. The 2 indels are strikingly less frequent than the 43 single-base substitutions. The general expectation that frameshifting indels generate a detectable mutant phenotype when arising in a protein-coding sequence reduces the chances of having missed other indels during the scoring of mutants. In addition, pQßRT, the RT-expressing plasmid used in this study, can complement *RT* deletions comprising up to 447 nt [36], which reduces the probability of having missed indels >1-nt long. The 39 transitions were almost 10-fold more frequent than the 4 transversions; if both transversions and transitions were to arise at equal frequencies among base-substitution pathways, the expected ratio would be 1∶0.5 (each site being able to generate two kinds of transversions and one kind of transition), a 20-fold difference from the observed ratio. Transitions, when ranked in decreasing order of observed numbers, were U→C (16)>G→A (10)>A→G (8)>C→U (5). The numbers of the four bases in the target decrease in the same order, U(175)>G(147)>C(139)>A(130), but this trend cannot quantitatively explain the normalized frequencies of mutated bases, which is 0.091>0.068>0.058>0.038. Thus, the intrinsic mutability of the four bases, presumably reflecting the error propensities of the Qß replicase, is likely to be the main determinant of the relative frequencies of observed mutations.

**Table 3:** Sequence changes in *RT*.

Mutation type	No.	Mispair^a^
Indels		
−1	2	
Transitions		
A→G	8	U•G
G→A	10	C•A
U→C	16	A•C
C→U	5	G•U
Transversions		
A→U	1	U•U
A→C	0	U•C
G→U	2	C•U
G→C	0	C•C
U→A	0	A•A
U→G	1	A•G
C→A	0	G•A
C→G	0	G•G
Total	45	

a Assuming mispairing at the second round of genome replication; the template base is underlined.

**Figure 3:**
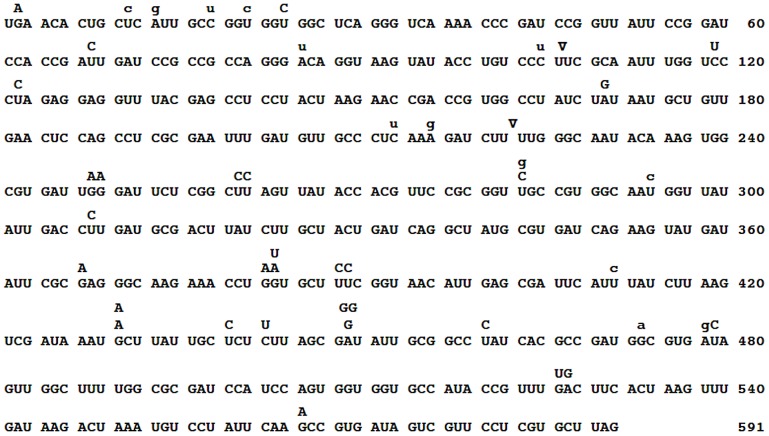
Spectrum of spontaneous Qß mutations. The main RNA sequence represents the codons of the positive strand of the mutational reporter, which includes the RT-coding gene from positions 1743–2333 of the Qß genome (*i.e.*, the RT-coding gene minus the portion that codes the Coat protein). The *RT* sequence begins with the UGA stop codon for the Coat protein, which at a frequency of 3% is translated as tryptophan to yield the whole RT protein. Mutations are indicated above the wild-type sequence. Primary base substitutions are in capital letters while secondary ones are in lower case. The two insertions are indicated by inverted triangles.

**Figure 4:**
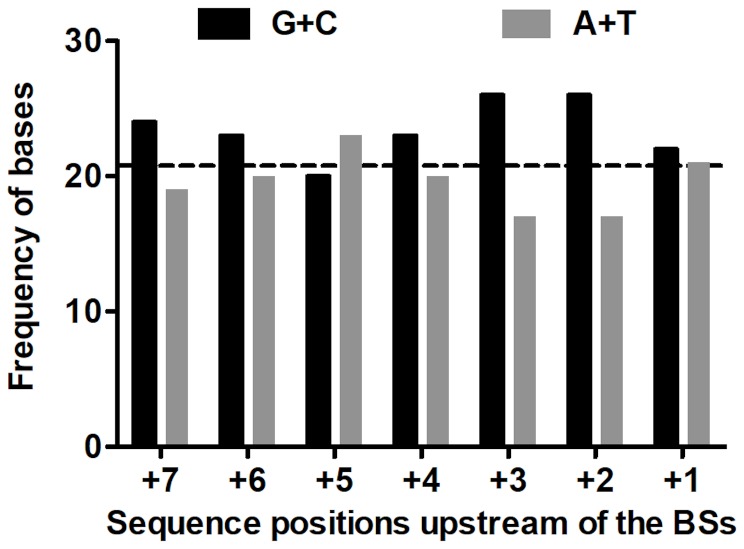
Frequencies of (G+C) and (A+T) upstream of base substitutions. The columns represent the absolute frequencies of G+C (in black) and A+T (in gray) in the 7 sequence sites immediately upstream of the 43 substitutions found in *RT*. The horizontal dashed line represents the average G/C expected per *RT* site based on 591 bases (GC content in *RT* = 0.484; 0.484×43 = 20.8). See text for details.

The mutations were widely distributed over the target (Figure 3). Because only 4 *RT* positions out of the observed 38 hosted more than one substitution, the spectrum is clearly far from saturation. Both indels arose within short homopolymeric runs, a common pattern in mutation spectra that presumably reflects misaligned primer-templates [37], [38]. The substitutions showed no correlation with their nearest neighbors either individually or as purines *versus* pyrimidines (analyses not shown). However, because a tendency towards enhanced mutability of any base within a G/C-rich sequence has been observed in both *E. coli* [39], [40] and the T-even coliphage RB69 [41], we also examined the base composition (G+C versus A+T) of the local sequence environments where substitutions were observed. G•C base pairs are more stable than A•U pairs, so that G/C-rich sequences might help to stabilize secondary structures containing hairpin loops, where unpaired bases may be more sensitive to oxidative damage. In addition, duplexes richer in G•C pairs may be slower to unwind, which might render replication more error-prone in currently unknown but perhaps generally applicable ways. A recent description of the structure of the Qß replicase [31] suggested that the replicating Qß genome (template+complement) forms a 6–7 base-pair duplex in the internal cavity of the replicase before both the single-stranded product and template exit the enzyme. Accordingly, we analyzed the base composition of the sequences six and seven bases upstream of the observed substitutions (Figure 4). Both the 6-mers and the 7-mers contain more (G+C) than expected from the target content of bases. The difference for the 6-mers has *P* = 0.059 and for the 7-mers has *P* = 0.034 (replicated G-test for goodness-of-fit, *P*-values for “pooled G”, G_P_, 1 df). Nevertheless, these small differences, combined with the homogeneity in base composition of the analyzed sequences, made the “total G” (G_T_) non-significant in both analysis (G_T_ = 0.409, 6 df, and G_T_ = 0.420, 7 df, for 6- and 7-mers, respectively). Overall, a larger sample of mutations would probably indicate more clearly the existence (or absence) of any effect of the G/C content of the local sequence on Qß-replicase error tendencies.

### The Mode of Genome Replication

To determine how mutations accumulate in the Qß (+)-strand progeny during replication and thus to estimate the rate of spontaneous mutation per genome replication in Qß, it is necessary to know the mode by which Qß produces its progeny during cell infection. Two distinct modes are possible. One is linear, wherein the infecting (+)-strand genome is used repeatedly as a template and then at least some of the resulting (−)-strand RNAs are each used repeatedly as templates; consequently, at the end of the infection cycle, each of the many (+)-strand progeny has experienced only two replications, from (+) to (−) and from (−) to (+). In this model, due to the many (+)-strand progeny contributed by the fewer (−)-strand templates, most replication errors will produce a single mutant during the second round of replication and only a small fraction of errors will generate a clone of mutants when a replication error occurs in the first round of replication and is further repeatedly copied in the second round [4]. The other mode is classical exponential replication, in which case the numbers of mutants recovered from single viral bursts display an exponential distribution [42]. Intermediate models combining linear and exponential replication in different proportions are also conceivable.

To determine which model best fits the distribution of mutants in Qß, two separate single-burst reversion tests were conducted (see Materials and Methods), one using the mutant RT_IN_ and the other using RT_SUB_ (described in Table 1). The tests involved plating cultures, containing bursts from infected RTH cells, onto NR16205 cells, aiming to deliver roughly 1 revertant-yielding burst on each of many plates. With RT_IN_, among the 250 cultures plated, six did not form plaques well and were discarded, and five had the following number of Qß plaques: 1254, 585, 345, 342 and 105. Because the plating efficiency of wt Qß with NR16205 is ≈1.5-fold lower than with the RTH strain, those numbers correspond to about 1881, 818, 518, 513 and 158 plaques, respectively. Since the frequency of *RT* ^+^ revertants in the starting RT_IN_ population was (4.63±1.50)×10^−6^ (mean ± SD, n = 5) and roughly 4560 infected cells were introduced into each of the 250 experimental cultures, the expected total number of revertant bursts produced by preexisting *RT* ^+^ phages was ≈5.3, in close agreement with the observed five large revertant bursts. The variation in numbers of revertants among these five bursts is consistent with the observed variation in burst sizes of wt Qß growing in the RTH host, 847±308 (mean ± SD, n = 3 one-step curves), and individual bursts with sizes from 300 to 3000 have been observed. In the case of RT_SUB_, among the 500 cultures assayed, one had to be discarded and another contained 935 plaques; in this case, the expected number of bursts from preexisting *RT* ^+^ revertants was 3.5. None of the cultures containing bursts attributable to preexisting *RT* ^+^ phages were included in the analyses.

The revertant distributions differed for the two mutants (Table 4). With RT_IN_, the distribution closely fitted a Poisson, supporting a linear mode of genome replication for Qß and strongly inconsistent with an exponential mode. With RT_SUB_, the distribution deviated significantly from a Poisson, showing an excess of plates containing ≥3 revertants. Even within a linear replication mode, however, these results may reflect either or both of two causes. The first is that different reversion pathways during the first and second rounds of replication will tend to have different rates at any particular site. With RT_IN_, the reversion target for the first replication consists of 5′-UCUUAAUUAAGU-3′ where the target is underlined and reversion to wild-type would probably occur by the deletion of UUAA or, perhaps less likely, by pseudoreversion by the loss of one base from any of the four homo-dinucleotides, producing a gene with one extra codon. Unusually, the second-replication target is 5′-ACUUAAUUAAGA-3′, which is identical to the first-replication target except for the outermost flanking bases. Thus, the two error rates might be very similar and the ratio of (+)-strand to (−)-strand products might have been large enough so that errors accumulated mostly during the second replication and the resultant revertant bursts were largely composed of clones of size 1. With RT_SUB_, however, reversion must have occurred along the available single-base-substitution pathways (up to 8 for a UAG stop codon, depending on the functional competence of the encoded amino acids), each of which differs between replications and which might therefore have displayed large rate asymmetries, which can easily exceed 100-fold in the case of DNA genomes [*e.g.*, 43]. The second cause is that the total number of copying events probably differ between (−)-strand and (+)-strand synthesis during cell infection; in Qß, for instance, the number of accumulated (+) strands was estimated to be about 10 times greater than the number of (−) strands [14], [44], so that revertant bursts of size 1 from the (+)-strand synthesis would then be more frequent than the larger bursts from the first rounds of replication. Notably, however, these larger bursts, once they appear, are expected to exhibit variable sizes that depend on, among other factors, the growth conditions [45], and that might therefore impact the observed distribution.

**Table 4:** Observed and expected distributions of *RT* ^+^ revertants in RT_IN_ and RT_SUB_ bursts.

	RT_IN_ a ^,^ b		RT_SUB_ a ^,^ b	
RT^+^ per plate	Observed	Expected^c^	Observed	Expected^c^
0	31	31.00	238	238.01
1	63	63.32	150	175.72
2	55	64.66	66	64.87
3	49	44.02	27	15.96
4	24	22.48	10	2.95
5	11	9.18	2	0.44
6	4	3.13	3	0.05
7	1	0.91	1	0.1
8	1	0.23	1	0
9	0	0.05	0	0
10	0	0.01	0	0
Total plates	239	239	498	498
Total plaques	510	488.13	446	367.67
*P* c	0.825		<0.001	

a Sets of 10 and 5 independent experiments were conducted with RT_SUB_ and RT_IN_, respectively. Because no significant differences were found in the observed distributions of revertants among the experiments of each set (G-test of independence, *P* = 0.3414 and *P* = 0.1103, with 16 and 27 df for the set of experiments conducted with RT_IN_ and RT_SUB_, respectively), the results of all the experiments comprising each set were pooled.

b Expected distributions of revertants were inferred using the *p*_0_ method, based on the fraction of plates displaying no revertants [47].

c *P* values represent the probability that the observed distributions fit the Poisson distributions as determined by G-tests for goodness-of-fit. See text for details.

### The Rate of Spontaneous Mutation

To estimate the rate of spontaneous mutation per genome replication (μ*_g_*) for a ribovirus, it is necessary to know (i) the mutation frequency *f*, (ii) the number of infection cycles *c* that elapse between the initial infection and the scoring of mutants, (iii) the average number of times *n* that each genome is replicated per infection cycle, (iv) the number of detectably mutable bases in the mutational target (*T*), and (v) the genome size (*G*). In the present case, *G* = 4217 nt, *c* = 1, and, from our results, *n*≈2. Although *T* = 591 *RT* bases for estimating the indel mutation rate (μ*_I_*), that number cannot be used when estimating the corresponding base-substitution rate (μ*_SUB_*) because, while nearly all indels are detectable, many substitutions fail to produce a mutant phenotype. Instead, μ*_SUB_* may be estimated from the number of substitutions that generate a stop codon (nonsense mutation) because, like indels, nonsense mutations are generally detectable. When considering nonsense mutations, *T* equals one-third of the number of paths in the mutational target that may generate a stop codon (one-third because each base can mutate by three different paths) [46]. In this study, 3 nonsense mutations were found among 7517 Qß isolates, and *T* = 66 paths leading to a stop codon in the *RT* target. Thus, *f_path_* = 3/(7517)(66) = 6.047×10^−6^, *f_SUB_* = 3*f_path_* = 1.814×10^−5^ per base, and μ*_SUB_* = *f_SUB_*/*cn* = 9.0704×10^−6^. Because 2 indels were found, *f_I_* = 2/(7517)(591) = 4.502×10^−7^, and μ*_I_* = *f_I_*/*cn* = 2.251×10^−7^. Hence, μ*_g_* = (μ*_I_*+μ*_SUB_*)*G* = 0.039.

In addition to the primary mutations detected by their phenotypes, some hitchhiking mutations were found. These secondary mutations may be used for an independent estimate of μ*_g_*, in which case *T* = 591 bases. A total of 9 secondary mutations (all base substitutions) were detected among 112 sequenced sub-isolates. (The remaining secondary mutations from the 13 described in Table 2 were observed in RT^−^ isolates lacking any detectable primary *RT* mutation and thus were excluded from these calculations.) Thus, *f_SUB_* = 9/(112)(591) = 1.36×10^−4^, μ*_SUB_* = *f_SUB_*/*cn* = 6.80×10^−5^, and μ*_SUBg_* = μ*_SUB_G* = 0.287. This value is greater than the corresponding value from the nonsense-mutation method by 7.4-fold and may, as discussed later, signal the impact of transient hypermutation.

Mutation rates can also be estimated for the reversion of the mutants RT_IN_ and RT_SUB_ using the results of the single-burst reversion tests. First, some definitions are needed: the number of cultures = *C*; the average number of infected cells per tube = *N*; the average burst size = *B*; the number of initial [(+)-strand→(−)-strand] copies = *c*_1_ with an error rate μ_1_ per copy; the number of succeeding [(−)-strand→(+)-strand] copies = *c*_2_ with an error rate μ_2_ per copy and a burst size *B* = *c*_2_ that ignores unpackaged genomes; and there are *n* = 2 two rounds of replication per infection. Then the average total number of mutational events per infected cell will be *c*_1_μ_1_+*c*_2_μ_2_; however, these components cannot be disentangled with our data, so we will assume that *c*_2_μ_2_≫*c*_1_≥_1_ (*e.g.*, most of the mutations are generated in the second replication, as indicated by the results from the single-burst reversion tests), in which case the average total number of mutations per infected cell will be *c*_2_μ_2_ = *B*μ_2_.

For a set of cultures of which some contain 0 mutants, the fraction of null tubes is e^−*m*^ where *m* is the average number of mutational events per culture [47]. The total number of replication events per culture≈*NB*, whence μ_2_≈μ = *m*/*NB*. For RT_IN_, the fraction of null tubes was 31/239, *m* = 2.04, *N*≈4560 infected cells per tube, and *B* = 328±93 (mean ± SD, n = 3 one-step curves), so that μ(RT_IN_) = (1.37±0.39)×10^−6^. For RT_SUB_, the fraction of null tubes was 238/498, *m* = 0.738, *N*≈35, *B* = 859±165 (mean ± SD, n = 3 one-step curves), and μ(RT_SUB_) = (2.46±0.47)×10^−5^. The ratio μ(RT_SUB_)∶μ(RT_IN_)≈18 which, given the indel sample size of 2, agrees well with the corresponding ratio of the two kinds of mutations (substitutions and indels) in the spectrum (43∶2≈22) or by rate (9.07×10^−6^)∶(2.25×10^−7^)≈40).

Another way to estimate these reversion rates is to use μ = *f*/2 but, as directly above, to assume that all detected mutations arose in the second round of replication, those arising in the first round being too infrequent to be readily observed, in which special case, μ = *f* as above. Here, *f* is simply the sum of all observed *RT* ^+^ revertants divided by all the Qß progeny in all tubes, *NBC*. For RT_IN_, the total number of revertants was 510 (Table 4), so that μ(RT_IN_) = 510/(4560)(328±93)(239) = (1.43±0.40)×10^−6^, a value close to the null-class value because of the excellent agreement between the observed distribution and the Poisson expectations (Table 4). For RT_SUB_, the total number of revertants was 446 (Table 4), so that μ(RT_SUB_) = 446/(35)(859±165)(498) = (2.98±0.58)×10^−5^, again a value close to the null-class value but slightly higher due to the occurrence of a small excess of plates with larger numbers of revertants compared to the expectations of the Poisson distribution (Table 4). Because the number of paths in which the RT_SUB_ mutated codon (UAG) may change producing an RT^+^ revertant is not known, the estimated μ(RT_SUB_) is an upper limit corresponding to 8 paths or 2⅔ substitutions.

## Discussion

### The Mutation Spectrum

We have obtained a spontaneous mutation spectrum for the RNA coliphage Qß using a cognate mutational target, the RT-coding gene minus the portion encoding the Coat protein. This 591-nt target generously samples the 4217-nt Qß genome, and the *RT* and genome base compositions are indistinguishable (G-test of independence, *P* = 0.9719, 3 df). The spectrum, based on 45 single-base changes, is a mixture of 32 primary mutations plus 11 secondary mutations found hitchhiking on some primary mutations, plus 2 single synonymous mutations (at target sites 18 and 294) arising during sequencing that showed no primary mutation. This spectrum has three defining characteristics. One is its strikingly low frequency of indels, only 2 among 30 *RT* mutants and 45 mutations, thus representing only about 4% of the total mutations, while in spectra from several DNA-based microbes (phages λ and T4, *E. coli, Saccharomyces cerevisiae*, and *Schizosaccharomyces pombe*), indels comprise about 40% of the mutations (average 41%, range = 27–59% [39], [46], [48]–[51]). Another characteristic is its unusually high transition∶transversion ratio (39∶4 = 9.75) compared to a random expectation of 1∶2 = 0.5. This transition bias contrasts with the transition∶transversion ratios observed for the same DNA-based microbes mentioned above (mean 0.87, range 0.08–1.67). Finally, normalized to target-base frequencies, the spectrum reveals a biased mutation tendency consisting of U→C>G→A>A→G>C→U> all transversions. Taking into account the dynamics of Qß genome replication with most mutations arising during the second round of replication, this mutation bias reflects a mismatch formation/extension bias in the template•progeny sense of A•C>C•A>U•G>G•U> transversions mismatches. This bias does not seem to reflect either cytosine deamination (which promotes C→U) or guanine oxidation (which promotes G→U), but rather the insertion of ionized, tautomerized, wobbled or *syn*-conformation bases.

Several other spectra of spontaneous riboviral mutations have been described previously.

- The first, using (+)-strand tobacco mosaic virus (TMV) and a target-complementation system *in trans*, reported a notable preponderance (24∶11) of indels over substitutions, similar numbers of transversions (6) and transitions (5), and a remarkably high frequency (9/23) of mutants with multiple mutations (“multiples”) [10]. These multiples may have arisen either because of transient hypermutability (which may also have been observed with Qß, as described below) or because the mutants were recovered after multiple sequential cell infections, estimated at *c*≈6. In this study, only mutants carrying lethal mutations were recovered. Thus, if many of the TMV base-substitutions were leaky, then, given *c*≈6, the high frequency of indels might have reflected their fully mutant phenotypes in contrast to the often leaky substitutions; however, although the TMV target sequence contained 115 paths to stop codons, no nonsense mutations were recovered in the small sample of substitutions. Perhaps some other factor impinged on this system, such as photodynamic mutagenesis.
- Another spectrum, obtained in a mutation-accumulation experiment using almost the entire genome as a mutational target for the dsRNA phage φ6, also exhibited a strong transition∶transversion bias (46∶5 = 9.2) and only one indel [6], although selection may have reduced the recovery of indels. In this phage, the parental genome can be written as [+/−]. Upon infection, the (−) strand is copied repeatedly into (+) strands, some of which are translated and others of which are encapsulated. Within the nascent viroid, the (+) strand is copied once to produce a [+/−] progeny particle. In this pathway, a mutation such as U→C can arise by an A•C mispair in the first round of replication, or by a U•G mispair in the second round. Both paths would produce clones of size 1 in a single-burst reversion test, the predominantly observed result [8], but the culpable mispair remains unknown. The observed mutation bias was A→G(14)≈C→U(13)≥G→A(10)≈U→C(9)>all transversions, or, when normalized to the numbers of target bases, A→G(0.0052)>C→U(0.0034)≥G→A(0.0027)≈U→C(0.0028). While this is approximately the opposite of the Qß bias, it could turn out to be very similar in terms of preferred mispairs depending on which mispairs generated the φ6 mutations.
- A (+)-strand tobacco etch potyvirus spectrum [11], obtained by means of a target-complementation system *in trans*, was probably contaminated with mutations arising during reverse transcription and PCR amplification of the isolated virus, with no way to sort out which mutations were of viral and which of processing origin because the existence of any relation between the detected mutations and a mutant phenotype was not examined.
- For the (+)-strand hepatitis C virus, mutation sampling *in vivo* based on deep sequencing of plasma samples from untreated patients revealed an approximately 75-fold ratio of transitions to transversions with all four transitions present at similar frequencies, whereas kinetic studies *in vitro* of the viral replicase revealed a strong bias in favor of G•U and U•G mismatches [52], although mismatch extension efficiencies were not much explored. This contrast resembles that seen in numerous reversion systems, which are typically limited to small numbers of templates that tend towards often strongly site-specific mismatching biases.

Taken together, the informative parts of these spectra indicate that riboviral mutation spectra differ from those characteristic of DNA viruses and cellular organisms in displaying many more transitions than transversions and an even smaller proportion of indels.

### The Mode of Genome Replication

With the aims of determining the way in which mutations accumulate during riboviral replication and estimating the rate of spontaneous mutation per genome replication, we investigated the mode in which Qß replicates its genome. Results from two independent single-burst reversion tests indicated that this mode is essentially linear, with the genome of each Qß progeny resulting from only two replications: from the original parental (+) strand to a (−) strand and then to a new (+) strand. Our results further suggest that most replication errors occur during the second round of replication, which in turn reveals the specific mismatches that produced the substitutions in the mutational spectrum.

An interesting result is that the distribution of *RT* ^+^ revertants deviated significantly from the expected Poisson distribution for RT_SUB_ but not for RT_IN_. With no reason to suspect that the two strains replicate their genomes differently, this discrepancy may reflect intrinsic differences between their reversion targets. The reversion target in RT_IN_ is the same in both rounds of replication, while reversion in RT_SUB_ may occur through up to 8 different single-base-substitution pathways in each round of replication. Thus, reversion rate asymmetries between the two rounds of replication may be anticipated for RT_SUB_, allowing some reversion to occur during the first round of replication and thus producing some revertant clones of size >1 during the second round.

**Figure 5:**
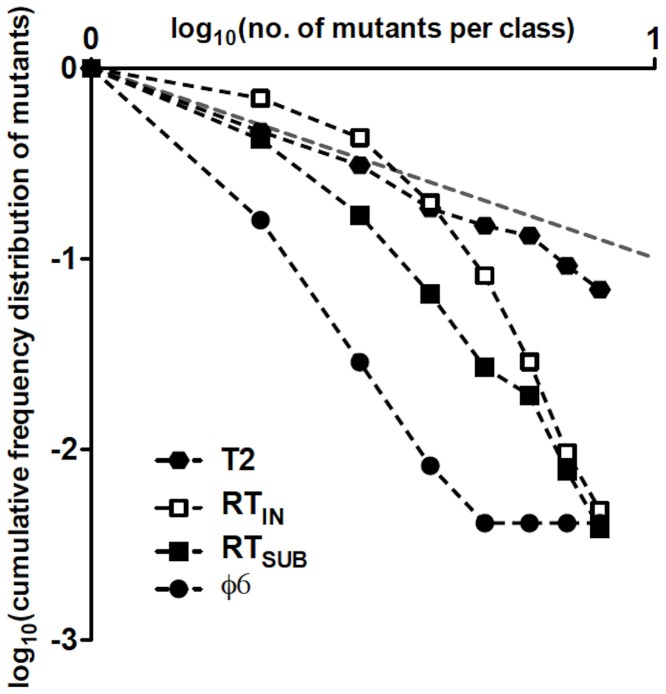
Double-logarithmic plots of the relative cumulative frequency distributions of mutants for T2, φ6, RT_IN_, and RT_SUB_. The relative cumulative frequencies were calculated from Table 3 in [42] for T2 and from Table 1 in [8] for φ6. The plot of a cumulative frequency distribution of phage mutants from a single-burst reversion test has a slope whose steepness indicates the mode of replication of the phage; a steep slope resulting from large differences in frequency between adjacent mutant classes indicates a linear mode of replication, while a shallow slope indicates an exponential mode of replication. In a log-log plot, where the relative cumulative frequencies of mutants and the clone-size classes have a similar range of values, a slope close to −1 (represented as a gray dashed line) will reflect an exponential mode of replication, while a linear mode of replication will show a slope<−1.

The mode of genome replication in riboviruses has been addressed in only a few instances. Using the single-burst reversion test, a predominantly linear mode was reported for the phage φ6 [8]. In that study, however, the observed distribution of mutants (*i.e.*, revertants) differed somewhat from the expected Poisson for a linear mode of replication, suggesting an exponential component in the replication dynamics that was estimated to generate ≈1% of the total progeny [8]. Such discrepancies between observed and Poisson distributions may occur because of sampling errors or the presence of a small exponential component. A way to discriminate among these is to plot the logarithm of the cumulative frequency distribution of observed mutants against the logarithm of the sizes of the mutant classes [53]. Figure 5 shows such plot for T2, φ6, RT_IN_, and RT_SUB_. In a log-log plot, exponential replication will display a linear relationship between the cumulative distribution of mutants and the number of mutants per class, with a slope close to −1. In linear replication, however, the plot will not be linear and the slope for the cumulative distribution will be steeper because most mutants arise in clones of size 1. In agreement with this reasoning, the data for T2, which replicates exponentially [42], exhibits a linear relationship with slope −1.20±0.03, based on the sum of the *r* and the *w* mutants and excluding all classes containing ≥16 mutants (*i.e.*, classes starting to approach the T2 burst size), while the plots for φ6, RT_IN_, and RT_SUB_ display nonlinear relationships.

In a recent report [9], the dynamics of (+)-strand and (−)-strand accumulation during cell infection were quantitatively analyzed for the (+)-strand RNA turnip mosaic virus using strand-specific quantitative real-time PCR. The results indicated that the virus replicates its genome in a mostly linear mode, in agreement with other quantitative results from *in silico* modeling of the optimal riboviral replication strategy in response to the error rate and the availability of resources, among other parameters [45], [54]. However, the continuous accumulation of turnip mosaic virus (−) strands throughout infection suggests that a purely linear mode of replication may have been unlikely; indeed, the occurrence of a trace of exponential replication was reported. While we cannot exclude a trace of exponential replication in the case of Qß, our results suggest that the RT_SUB_ revertant distribution may depart from a Poisson distribution mostly due to asymmetries in the reversion rates at the first and the second rounds of replication.

Overall, the empirical data gathered to date on the riboviral mode of replication indicate that, regardless of the single- or double-stranded genome structure of the virus, the strategy of preference is mainly linear. The advantages that this mode of replication may confer to riboviruses over an exponential mode have been evaluated previously (*e.g.*, [8]).

### The Spontaneous Mutation Rate

Our results provide several independent estimates of the spontaneous Qß mutation rate per genome replication (μ*_g_*). The first is based mainly on the small set of three nonsense mutations detected among 30 *RT* mutants. Because many base substitutions do not produce a detectable phenotype, the estimation of the μ*_SUB_* fraction of μ*_g_* = (μ*_SUB_*+μ*_IN_*)*G* from the frequency of nonsense mutations is a preferred method because nonsense mutations are highly detectable and their target size is easy to determine from the codon composition of the mutation target. However, this method has two drawbacks: nonsense mutations are typically a small fraction of all substitutions, so that sufficient mutants must be harvested and sequenced for a reliable estimate [46]; and no substitutions to C (in the coding strand) can generate a stop codon, so that the average rate per base from the nonsense-generating pathways must be assumed to apply to all pathways. Using the nonsense-mutation method and adding the small component due to indel mutagenesis, the Qß genomic rate was estimated to be μ*_g_* = 0.039 per replication or about 0.08 per infection cycle. For this nonsense-based estimate of μ*_g_*, *RT* mutants were collected after one-step growth of wt Qß, so that *c* = 1 in the calculations. While some prior *RT* mutations may have arisen during the growth of the wt Qß stocks in non-complementing NR16205 lawns, lethal indels and nonsense mutations would have been subjected to strong negative selection. In some riboviruses, mutants bearing lethal mutations can grow in the presence of complementation *in trans* provided by a plasmid (*e.g.*, this study) or by a gene inserted into a host chromosome (*e.g.*, [10]), and thus it may be assumed that complementation can also be provided by a co-infecting wild-type phage, which means that even *de novo* Qß mutants carrying an *RT* ^−^ mutation may have expanded during the growth of the original wt Qß stocks, rendering 1<*c*≤3. In the case where only one *RT* ^−^ mutant and one wild-type co-infect the same host cell and up to 50% of the resulting progeny are *RT* ^−^ mutants, the consequent selection coefficient (*s*) of the *RT* ^−^ mutant will be 0.50 per infection cycle. Applying the method described in Burch *et al*. [6] to estimate the effect of selection within the plaque and considering μ*_g_* = 0.039, the probability of loss of an *RT* ^−^ mutant with *s*≥0.50 arising in the first infection cycle of wt Qß on a host lawn would be ≥42% at the end of the growth phase (Figure S1). However, previous reports [22], [55] indicate that co-infection by distinct Qß mutants and consequent complementation occur at low to undetectable frequencies. Even if any *RT* ^−^ mutation arose during the last cycle of growth in NR16205 lawns, the small fraction of each wt Qß isolate used to establish the one-step lysates further reduced the frequency of preexisting *RT* ^−^ mutations in our starting wt Qß populations.

The second method for estimating μ*_g_* is based on the single-burst reversion tests. Here, the mutation rate is based on the null-class method [47]. For RT_IN_, μ_target_ = 1.37×10^−6^ and the number of mutating bases in the four-base duplication may be taken as 4 (although it may be argued to be 1); then μ*_INg_* = (4217/4)(1.37×10^−6^) = 0.0014. For RT_SUB_, μ_target_ = 2.46×10^−5^ and the potential number of mutating bases is 3; then μ*_SUBg_* = (4217/3)(2.46×10^−5^) = 0.035. The sum of the indel and the substitution rates is μ*_g_* = 0.036 (0.041 if the indel reversion target size is taken as 1), a value (perhaps deceptively) close to that of μ*_g_* = 0.039 calculated from the spectrum.

The third method for estimating μ*_g_* applies only in cases where the mutations are not required to produce a mutant phenotype, which can arise when a target is sequenced without regard to phenotype (provided the mutation is not a dominant lethal) or when, as in the present case, hitchhiker mutations arise secondarily to and in combination with a driver mutation, the target then consisting of the entire sequence of the mutation reporter. Hitchhikers could arise during any of the roughly 3 infection cycles that generate a Qß plaque but, in order to be detected, most would have to arise in the first cycle with μ*_g_* = 0.287. This value may be an underestimate because the low ratio (6/7) of missense mutations to synonymous mutations among the secondary *RT* mutations suggests significant selection against missense mutations during plaque growth even in RTH lawns; if the hitchhikers were a random set, then missense mutations would comprise about ¾, or 9.75, of 13 substitutions. In fact, RT_IN_ has an average burst size in RTH cells that is 2.9-fold smaller than that of wt Qß, a difference that implies a selection coefficient of *s*≈0.65 per infection cycle. An *RT* mutant with *s*≥0.65 arising with μ*_g_* = 0.287 in the first infection cycle on a RTH lawn would have a ≥40% probability of being lost by the end of the growth phase (Figure S1). Thus, μ*_g_* = 0.287 estimated from hitchhikers might be significantly underestimated.

The presence of more mutants with multiple mutations than expected from a random distribution is remarkably widespread among DNA and RNA genomes and is probably more often due to transient hypermutation caused by some temporary perturbation of replication-fidelity factors than due to mutator mutations [56], [57]. A notable example is the considerably higher frequency of mutations than expected among mutants already carrying a driver mutation produced by the replicase of the DNA phage RB69 [56]. However, because the Qß replicase gene occupies 42% of the genome and the estimated μ*_g_* is high, we considered that some mutants might have arisen in a mutator background and then gone on to produce hitchhikers at an increased frequency. Therefore, we examined whether the gene encoding the ß subunit of the Qß replicase harbored mutations in the 28 *RT* mutants carrying detectable primary mutations and their four parental wild-types. We observed three T→C substitutions (at ß-subunit position 75 of mutant RT32, position 550 of RT42, and position 1668 of RT20) but all were synonyms, so that replicase mutators were apparently not impacting our set of *RT* mutations. Instead, the excess of secondary mutations among our *RT* mutants may have arisen by the action of an abnormal replicase ß subunit produced by an error of translation or protein conformation.

Among the three values, our best estimate of μ*_g_* was obtained by the nonsense-mutation method. While the rate obtained from the reversion tests was similar, its accuracy depends on the extent to which the two mutants fairly sample the whole genome, and the similarity may have been fortuitous. Both selection and transient hypermutation may have played an important role in the production of the *RT* mutations considered in the third method. Unfortunately, even our favored μ*_g_* estimate is based on small samples of mutations (3 nonsense and 2 indels), which enlarges the margin of potential sampling error. When our first and second μ*_g_* estimates are combined with the fraction (0.4) of random mutations that are lethal for the (−)-strand-RNA vesicular stomatitis virus [58], (0.075 mutations per infection cycle ×591 nt per target ×7517 targets tested ×0.4 of mutations detectable)/4217 nt per genome = 32 *RT* mutants, in close agreement with the 30 observed and providing modest further support for μ*_g_*≈0.04.

While the mutation rates per genome replication estimated here and reported for TMV [10] and phage φ6 [6] are all in the neighborhood of 0.04, rates for mammalian riboviruses center around 0.7 and display a wide range [7]. However, the latter rates were based on tiny targets often consisting of a single base or pathway and may have been reported because they were large and thus more easily measured; alternatively, as has been frequently suggested, immune surveillance in mammals may drive higher mutation rates. It is interesting that while the mutation frequency can be increased over the background with nitrous acid by up to 80-fold in tobacco mosaic virus with retention of some viability [59], it can be increased only about 2.5-fold in poliovirus and vesicular stomatitis virus before extinction begins [60], suggesting that mammalian riboviruses do indeed sustain mutation rates substantially higher than those of phage and plant riboviruses. Finally, although co-infection and complementation do not seem to occur at a detectable frequency with Qß, it may occur with other riboviruses, perhaps somewhat elevating mutant frequencies and thus causing mutation rates to be overestimated.

## Materials and Methods

### Plasmids, Bacterial Strains, and Growth Media

Plasmids and bacterial strains are listed in Table 1. All three pQß plasmids express the indicated Qß components constitutively and have been described [35], [36], [61]. The RT_IN_ mutant carries a tandem duplication of 2158-UUAA-2161 that corresponds to 416–419 in the target sequence. Cell transformations with the plasmids were performed using CaCl_2_ [62]. Unless otherwise indicated, RTH cells were grown in Luria-Bertani medium (LB) supplemented with 2 mM CaCl_2_ and 100 µg/ml trimethoprim (TMP), while NR16205 cells were grown in LB containing 15 µg/ml tetracycline. Cells and phages were plated using LB bottom agar with 2.0% Bacto agar. The top agar was always made up in distilled water. For counting plaques or scoring mutants, the top agar contained 0.4% Sigma-Aldrich Noble agar; for other uses, it contained 0.8% Bacto agar. All growth was at 37°C.

### One-Step Growth

NR16205 cells were transformed with pQßm100 (which expresses wt Qß) and plated on NR16205 lawns to yield wt Qß plaques, which were independently harvested into tubes containing 1 ml D broth (0.2% Bacto tryptone, 0.5% NaCl) and 25 µl of chloroform. For one-step growth curves in RTH cells, 10 µl of phage suspension from a wt Qß isolate was mixed with 1 ml of cells at OD_600_≈0.5 (10^8^ cells/ml) at a multiplicity of infection (MOI)≈0.01 for 20 min at room temperature, centrifuged to remove non-adsorbed phages, resuspended, and serially diluted in LB+TMP. Samples diluted 10^3^- and 10^5^-fold were held for 3 h at 37°C with gentle shaking, and 100-µl aliquots were removed from each dilution every 10 min and plated with RTH cells. Plates were incubated overnight and the follow-on titers were used to estimate Qß densities over time. Three one-step growth experiments were conducted in parallel for each wt Qß isolate used to generate one-step lysates. Visual inspection of the resulting curves sufficed to determine the time (≈75 min) for Qß to complete one infection cycle in RTH cells. These one-step curves were also used to estimate the burst size of wt Qß in RTH cells according to the protocol detailed for RT_IN_ and RT_SUB_ (see Table S1).

### Qß Replication Mode

The distribution of *RT* ^+^ revertants among *RT* ^−^ bursts was monitored as in a previous study [8] using two different *RT* ^−^ mutants (RT_IN_ and RT_SUB_ as described in Table 1). Preliminary measurements provided their burst sizes (Figure S2, Table S1) and revertant frequencies, which are needed to conduct the burst experiments. Ten and five independent experiments were carried out with RT_SUB_ and RT_IN_, respectively, and ≈500 *RT* ^+^ revertants were scored per mutant. In each experiment, ≈10^6^ phages were added to 1 ml of RTH cells at OD_600_≈0.5. After 20 min of adsorption at room temperature, the mixture was centrifuged for 1 min at 8,000 *g* and the pellet was resuspended in 1 ml LB broth. From the supernatant, 100 µl were collected to estimate the amount of non-adsorbed phages. The resuspended pellet was further diluted and 50 aliquots of 100 µl each were distributed into individual tubes, where infection was allowed to continue for ≈75 min at 37°C and then stopped with 15 µl of dichloromethane. Lysates were aerated for 30 min at 37°C to allow the dichloromethane to evaporate and their entire volumes were then independently plated on NR16205 lawns. The observed distributions of *RT* ^+^ revertants were compared to the expected Poisson distributions using G-tests for goodness-of-fit.

### Isolating and Sequencing Spontaneous *RT* Mutants

To limit the number of infection cycles to one before seeking spontaneous mutants, *RT* mutants were scored among the progeny of one-step growth of wt Qß in RTH cells. RTH cells were infected with wt Qß (MOI≈0.01) as above and one-step lysates were recovered by adding chloroform after 75 min of growth. Samples from the lysates were plated on RTH lawns at ≈70 plaques per plate and well-isolated plaques were independently sampled into 96-well plates containing 0.6 ml D-broth per well (reserving six un-inoculated wells as cross-contamination controls). For each of four independent lysates, three different rounds of 630 isolations each were performed. In each round, a control plate containing 8 wt and 82 RT_IN_ isolates was also established to confirm the ability of RTH cells to complement *RT^−^* mutants and the inability of any RTH cells remaining in the isolates to grow in LB supplemented with tetracycline. Isolates were spotted in parallel on lawns of NR16205 and RTH cells using a 6×8-array replica plater. After a few losses, a total of 7517 plaques were tested. Isolates that grew poorly in NR16205 cells were re-tested in both bacterial strains and the RT-coding genes of two independent sub-isolates per putative *RT* mutant were sequenced. After this first round of sequencing, two additional sub-isolates as well as the original isolate were sequenced for each verified *RT* mutant. The original wt Qß isolate used to develop each lysate and two sub-isolates of it were also sequenced.

Plate lysates were prepared from RTH cells (0.25 ml at OD_600_≈0.5) mixed with phages at MOI≈0.1 in Noble top agar. After overnight incubation, the plates were covered with 7 ml of SM buffer with gelatin [62] and were gently rocked for 30 min. The SM buffer was recovered and 100 µl of chloroform were added to each sample. Cell debris was removed by centrifugation at 12,000 *g* for 10 min. The supernatant was supplemented with polyethylene glycol (PEG 8000) to 10% w/v and NaCl to 1 M, incubated for 1 h on ice, and centrifuged at 3,000 *g* for 15 min at 4°C [63]. The pellets were resuspended in 2 ml of 10 mM MgSO_4_, 10 mM Tris-HCl, pH 8, and the resulting concentrated phages were used as sources for RNA purification. Phage RNA was isolated using the QIAamp Viral RNA Mini Kit. From the extracted RNAs, 10 µg were then treated with DNase I (New England BioLabs) to degrade residual host DNA. The DNase-treated product was purified using the RNAeasy Mini Kit. From the purified RNA, 1 µg was subjected to reverse transcription with the Omniscript RT Kit and about 25 ng of the RT product was amplified with PfuTurbo DNA polymerase (Stratagene). PCR products were confirmed by agarose gel electrophoresis, purified with the QIAquick PCR Purification Kit, and sequenced using BigDye Terminator v3.1 (Applied Biosystems). All kits were purchased from Qiagen and were used according to the manufacturer's recommendations. Sub-isolates showing secondary mutations were subjected to a second round of RT, amplification and sequencing. The primers utilized in the RT, PCR, and sequencing reactions and the PCR cycling parameters are listed in Table S2.

### Statistical Analyses

The *RT* and genome base compositions were compared using the G-test of independence. This test was also applied to compare the observed distributions of *RT* ^+^ revertants among the single-burst reversion tests conducted with each of two different *RT* ^−^ mutants, RT_SUB_ and RT_IN_. The G-test for goodness of fit was used to compare the observed and expected Poisson distributions of *RT* ^+^ revertants among *RT* ^−^ single-bursts, and the replicated G-test for goodness of fit was applied to compare the G+C content of the local sequence environment (six to seven bases upstream) of the base substitutions observed in *RT* with the expected content according to the base composition of the whole gene. When applying this last test, each upstream position (from +1 to +6 or +7) was considered as an independent replicate. All tests were performed as per Sokal and Rohlf [64].

## Supporting Information

Figure S1 Probability that a new *RT* mutation with selection coefficient *s* is lost during plaque growth in a host lawn. The black line represents the estimations for μ*_g_* = 0.039 while the gray line represents the estimations for μ*_g_* = 0.287.
(TIF)

Figure S2 One-step curves for RT_IN_ and RT_SUB_. Three independent curves were obtained per *RT* mutant. In all cases, the first bursts appeared between 30 and 40 min after infection. If the 20 min allowed for adsorption are also considered, it means that Qß requires a minimum of 50 to 60 min to complete an infection cycle in RTH cells. Thus, if any of the progeny released in the first bursts would have immediately infected a new cell, the first second-generation bursts might have been expected 100 min after the first infection. Indeed, the curves show a slight increase of Qß density at that time. For this reason, values collected 100 min after infection (empty symbols) were not considered in the curve-fitting analyses conducted to characterize RT_IN_ and RT_SUB_ single-burst dynamics. Squares represent outliers that were automatically excluded from the analyses, and the resulting fitted curves are shown as dashed curves. PFU = plaque-forming units. See Table S1 for additional information.
(TIF)

Table S1 Parameters of the sigmoidal curves fitted to the RT_IN_ and RT_SUB_ one-step curves shown in Figure S2.
(DOC)

Table S2 Primers and PCR cycling parameters for amplifying and sequencing.
(DOC)
