# Supplementary figures and images for: The Three Faces of Riboviral Spontaneous Mutation: Spectrum, Mode of Genome Replication, and Mutation Rate
Source: PLoS Genet. 2012 Jul 26;8(7):e1002832. doi: 10.1371/journal.pgen.1002832 (PMC3405988; doi:10.1371/journal.pgen.1002832)

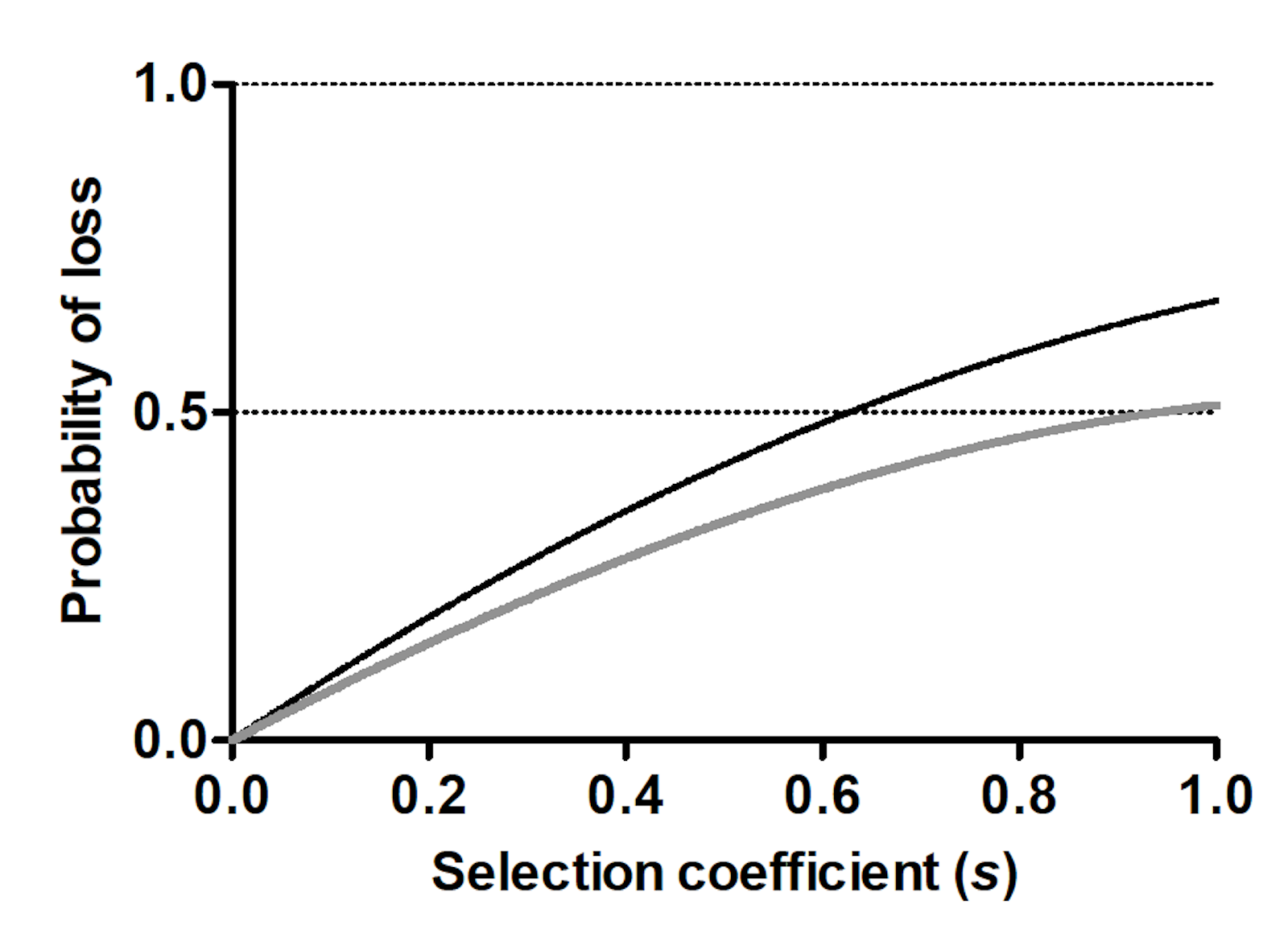

Supplement: Figure S1 — Probability that a new RT mutation with selection coefficient s is lost during plaque growth in a host lawn. The black line represents the estimations for μg = 0.039 while the gray line represents the estimations for μg = 0.287. (TIF) [file pgen.1002832.s001.tif]

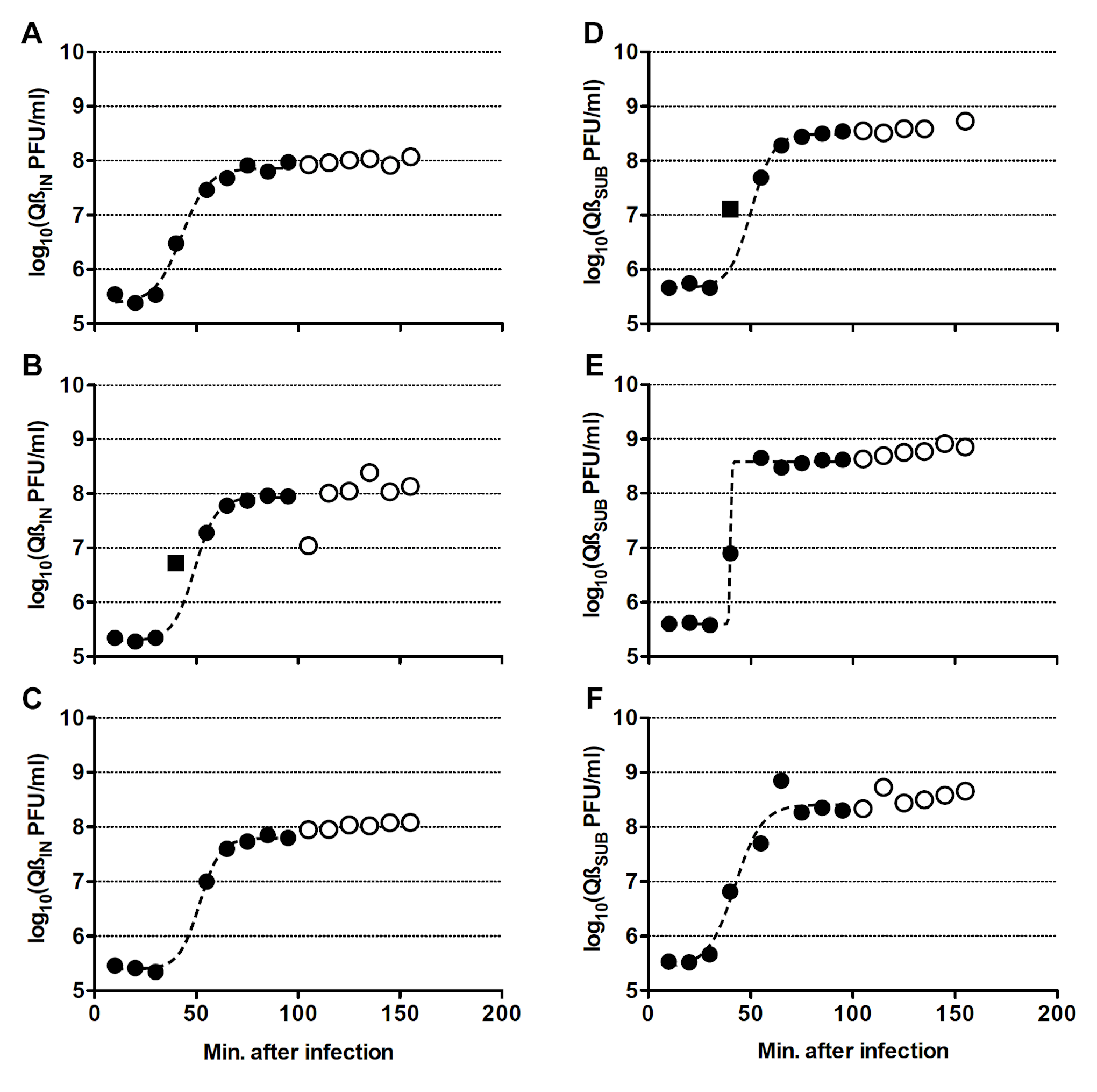

Supplement: Figure S2 — One-step curves for RTIN and RTSUB. Three independent curves were obtained per RT mutant. In all cases, the first bursts appeared between 30 and 40 min after infection. If the 20 min allowed for adsorption are also considered, it means that Qß requires a minimum of 50 to 60 min to complete an infection cycle in RTH cells. Thus, if any of the progeny released in the first bursts would have immediately infected a new cell, the first second-generation bursts might have been expected 100 min after the first infection. Indeed, the curves show a slight increase of Qß density at that time. For this reason, values collected 100 min after infection (empty symbols) were not considered in the curve-fitting analyses conducted to characterize RTIN and RTSUB single-burst dynamics. Squares represent outliers that were automatically excluded from the analyses, and the resulting fitted curves are shown as dashed curves. PFU = plaque-forming units. See Table S1 for additional information. (TIF) [file pgen.1002832.s002.tif]
