# Supplementary material for: The Three Faces of Riboviral Spontaneous Mutation: Spectrum, Mode of Genome Replication, and Mutation Rate
Source: PLoS Genet. 2012 Jul 26;8(7):e1002832. doi: 10.1371/journal.pgen.1002832 (PMC3405988; doi:10.1371/journal.pgen.1002832)
Supplement: Table S1 — Parameters of the sigmoidal curves fitted to the RTIN and RTSUB one-step curves shown in Figure S2. (DOC) [file pgen.1002832.s003.doc]

**Table S1.** Parameters of the sigmoidal curves fitted to the RT_IN_ and RT_BS_ one-step curves shown in Figure S2.

| Qß strain | Graph | x0 | xd | y1 | y2 | R^2^ | Burst size |
| --- | --- | --- | --- | --- | --- | --- | --- |
| RT_IN_ | A | 42.78±1.69 | 6.50±1.47 | 5.38±0.12 | 7.86±0.08 | 0.9900 | 301 |
|  | B | 49.20±0.97 | 5.31±0.76 | 5.30±0.03 | 7.93±0.03 | 0.9994 | 432 |
|  | C | 51.61±1.14 | 5.05±1.28 | 5.40±0.04 | 7.80±0.04 | 0.9980 | 251 |
| RT_BS_ | D | 50.23±1.11 | 5.35±1.02 | 5.67±0.04 | 8.49±0.04 | 0.9989 | 668 |
|  | E | 40.07 | 0.26 | 5.60±0.05 | 8.58±0.03 | 0.9988 | 916 |
|  | F | 42.42±3.09 | 6.61±2.73 | 5.43±0.27 | 8.41±0.18 | 0.9676 | 953 |

Graph letters refer to each of the curves shown in Figure S2. The parameters x0, xd, y1 and y2 represent, respectively, the inflexion point, the slope, the lowest, and the highest y limits of the curve as defined by y = y1 + (y2 – y1)/(1 + e^(x0 – x)/dx^), and R^2^ (0 ≤ R^2^ ≤1) is the goodness-of-fit for the curve. Burst sizes were estimated from the difference between y2 and y1.
