# Supplementary material for: The Three Faces of Riboviral Spontaneous Mutation: Spectrum, Mode of Genome Replication, and Mutation Rate
Source: PLoS Genet. 2012 Jul 26;8(7):e1002832. doi: 10.1371/journal.pgen.1002832 (PMC3405988; doi:10.1371/journal.pgen.1002832)
Supplement: Table S2 — Primers and PCR cycling parameters for amplifying and sequencing. (DOC) [file pgen.1002832.s004.doc]

**Table S2.** Primers and PCR cycling parameters for amplifying and sequencing.

| **RT and PCR reactions** | | | |  | **Sequencing** | |
| --- | --- | --- | --- | --- | --- | --- |
| Primer | Primer sequence | PCR | Product |  | Primer | Primer sequence |
| name^a^ | (5′→3′) | parameters^b^ | length (bp) |  | name^a^ | (5′→3′) |
| RT_AmpR | acttctcccaggcaacagctt | Annealing: | 1415 |  | RT_SeqF1 | ATTAACCCAACGCGTAAAGC |
| RT_AmpF | GCAATCTTCCGTTCGCTACAC | 68 ºC, 1 min |  |  | RT_SeqF2 | CGCAGTATAGTACCGATGAGG |
|  |  | Extension: |  |  | RT_SeqF3 | CGGCTTAGTTATACCACGTTC |
|  |  | 72 ºC, 2 min |  |  | RT_SeqR1 | GAGAGAGTTACGCGAAGATGC |
|  |  |  |  |  | RT_SeqR2 | GTCGCATCAAGGTCAATATAA |
|  |  |  |  |  | RT_SeqR3 | TCGGTACTATACTGCGTGAAC |
|  |  |  |  |  |  |  |
| ßsub_AmpF1 | CCGATGGCGTGATAGTTG | Annealing: | 1887 |  | ßsub_SeqF1 | GTGCCATACCGTTTGACTTC |
| ßsub_AmpR1 | TCGTGCCCTGGAAGACC | 65 ºC, 1 min |  |  | ßsub_SeqF2 | CTCGGGCTTTGAAGTATGTTTTAG |
|  |  | Extension: |  |  | ßsub_SeqF3 | GGAAGTTTTTAAGTATGTTGGTTT |
|  |  | 72 ºC, 2 min |  |  | ßsub_SeqR1 | CCGTAGGGTGCCAGAAC |
|  |  |  |  |  | ßsub_SeqR2 | AAACCAACATACTTAAAAACTTCC |
|  |  |  |  |  | ßsub_SeqR3 | CTGATATCGAAATGTGTAGAAGC |
|  |  |  |  |  |  |  |
| ßsub_AmpF2 | GCGGCAAGCACTACTATTCT | Annealing: | 655 |  | ßsub_SeqF4 | ACCTCTTCTCGCGTTGTCTCT |
| ßsub_AmpR2 | GATCCCCCTCTCACTCGT | 62 ºC, 1 min |  |  |  |  |
|  |  | Extension: |  |  |  |  |
|  |  | 72 ºC, 1 min |  |  |  |  |

^a^The RT primers were used for the mutation reporter and the ßsub primers were used for the Qß replicase.

^b^The remaining parameters were as specified by the PfuTurbo DNA polymerase manufacturer’s instructions. In all cases, the second segment of the PCR was cycled 35 times.
